# A brief overview of antitumoral actions of bruceine D

**DOI:** 10.37349/etat.2020.00013

**Published:** 2020-08-31

**Authors:** Zi Wayne Sin, Vipul Bhardwaj, Amit Kumar Pandey, Manoj Garg

**Affiliations:** 1 Department of Biological Sciences, National University of Singapore, Singapore 117600, Singapore; 2 Amity Institute of Molecular Medicine and Stem cell Research (AIMMSCR), Amity University Uttar Pradesh, Noida 201313, India; 3 Amity Institute of Biotechnology, Amity University Haryana, Manesar, Haryana 122413, India; National University of Singapore, Singapore

**Keywords:** Cancer, bruceine D, epithelial-to-mesenchymal transition, cancer stem cells, metastasis, apoptosis, anti-inflammatory, PI3K/AKT/ERK pathway

## Abstract

Cancer remains the second leading cause of mortality globally. In combating cancer, conventional chemotherapy and/or radiotherapy are administered as first-line therapy. However, these are usually accompanied with adverse side effects that decrease the quality of patient’s lives. As such, natural bioactive compounds have gained an attraction in the scientific and medical community as evidence of their anticancer properties and attenuation of side effects mounted. In particular, quassinoids have been found to exhibit a plethora of inhibitory activities such as anti-proliferative effects on tumor development and metastasis. Recently, bruceine D, a quassinoid isolated from the shrub *Brucea javanica* (L.) Merr. (Simaroubaceae), has come under immense investigation on its antineoplastic properties in various human cancers including pancreas, breast, lung, blood, bone, and liver. In this review, we have highlighted the antineoplastic effects of bruceine D and its mode of actions in different tumor models.

## Introduction

Cancer can result from uncontrolled cellular proliferation and growth that forms an abnormal mass of tissue known as a tumor. It can be caused by a plethora of risk factors such as genetic mutation due to exposure to radiation or carcinogens, epigenetics, viral infection, and reactivation [1–5]. According to the World Health Organisation, cancer is the second leading cause of global mortality in 2018 [6]. It is therefore not surprising that cancer research remains at the cynosure of the scientific community. Chemotherapy and radiotherapy are commonly administered for the treatment of human malignancies with temporary relief and adverse side effects such as nausea, vomiting, and hair loss, drastically diminishing the quality of life of cancer patients [3, 7]. In the past decade, research on natural bioactive compounds derived from plants and microbes have been gaining traction [8–13]. They can attenuate the side effects of conventional cancer treatment [14]. Importantly, these natural compounds possess anti-cancer effects [14–22]. In the field of cancer, natural products or their derivatives constitute approximately 49% of small molecule drugs approved by the United States Food and Drug Administration (FDA) from the 1940s to the end of 2014 [23]. As of 2019, 7 out of 48 newly approved drugs by the US FDA were inspired based on natural products [24]. While these drugs were not naturally derived compounds *per se*, it still highlighted the importance of natural bioactive compounds in novel drug development.

*Brucea javanica* (L.) Merr. (Simaroubaceae) is a shrub that is widely distributed in most of Asia and Australia [25]. The fruits and seeds of *Brucea javanica* have long been used as Chinese medicine in treating various diseases like dysentery, malaria, and inflammatory diseases [25, 26]. Of particular interest are the quassinoids isolated from the fruits of *Brucea javanica*. There are currently 52 known quassinoids such as brusatol, dehydrobruceine D, and bruceine D (BD) isolated from the fruits and seeds of *Brucea javanica* [27]. These quassinoids have been shown to exhibit a wide range of inhibitory effects, including anti-viral effects against plant-based viruses like tobacco mosaic virus and potato virus Y to anti-proliferative and cytotoxic effects on various tumor cells like lung cancer tumors and breast cancer tumors [28–34]. In this review, we will specifically be summarizing the antineoplastic effects of BD on various cancer cell lines from the current literature.

## Chemistry of BD

BD is a quassinoid with molecular formula C_20_H_26_O_9_. Many quassinoids have been found to exhibit tumoricidal activity and anticarcinogenic properties, with BD having found to exhibit cytotoxic effects and anti-proliferative effects against pancreatic cancer, breast cancer, lung cancer, leukemia, osteosarcoma, and hepatocellular carcinoma [35]. The structure-activity relationship of the inhibitory effects of some quassinoids, including BD, has only been elucidated very recently. Here, we will give particular focus to the structure-activity relationship of the antineoplastic effects of quassinoids. The substitution of oxygen-groups, methyl or -CH_2_OH, glycosyl, α, β-unsaturated ketone group, and olefenic bond was found to be pertinent in influencing the antineoplastic actions of quassinoids. Various modifications to the aforementioned chemical groups have been investigated and the extent of the anti-proliferative activity of the tested quassinoids against pancreatic cancer and breast cancer cell lines varied accordingly [29, 30, 36, 37].

## Antineoplastic effects of BD in human malignancies

### Anti-proliferative and pro-apoptotic effects of BD

**Figure 1.**
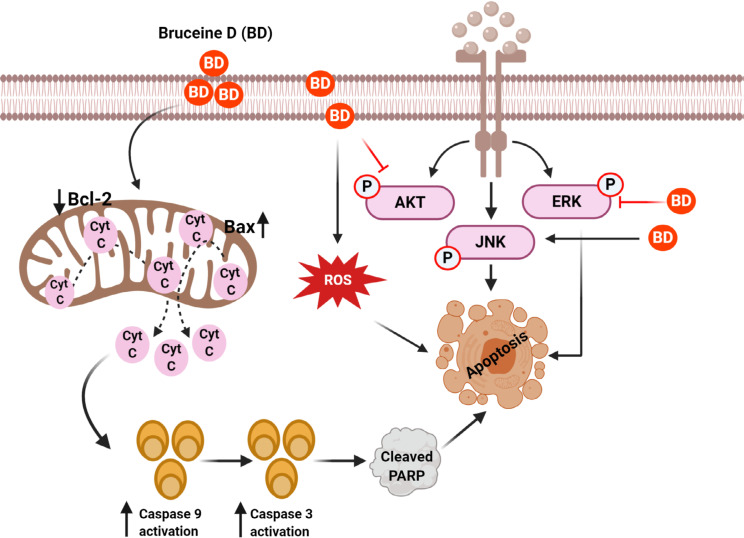
Mechanisms of BD for its anticancer effect in human cancers. BD treatment suppressed the AKT/ERK and activate the JNK signaling cascades to induce apoptosis of cancer cells. BD caused apoptosis of cancer cells by regulating ROS and mitochondrial proteins

It is known that dysregulation of cell cycle leading to uncontrolled cell proliferation and apoptosis evasion are hallmarks of all cancer cell types [29, 38–41]. Indeed, through various cell proliferation assays like Sulforhodamine B assay, MTT assay, and Cell Counting Kit 8 assay, BD has been established to exhibit anti-proliferative properties against pancreatic cancer cells (PANC-1, SW1990, CAPAN-1) [29, 37], lung cancer cells [A549, NCI-H292, non-small cell lung cancer (NSCLC) H460] [28, 42, 43], chronic myeloid leukemia (K562) [44], breast cancer cells (MDA-MB-231) [30], hepatocellular carcinoma cells (Bel7404, HepG2, Hep3B, Huh7, PLC) [45, 46], and osteosarcoma cells (MNNG/HOS, U-2OS, MG-63, Saos-2) [47]. In all of the investigated cancer cell lines, there was increased activation of pro-apoptotic proteins like B-cell lymphoma 2 (Bcl-2) associated protein (Bax) and Bak and downregulation of anti-apoptotic proteins like Bcl-2 and myeloid cell leukemia 1 (Mcl-1) [48], all of which are tightly linked to cellular proliferation and apoptotic pathways like phosphatidylinositol 3-kinase (PI3K)/protein kinase B (AKT)/mammalian target of Rapamycin (mTOR), c-Jun N-terminal kinase (JNK), mitogen-activated protein kinases (MAPK), and canonical Wnt signaling pathways (Figure 1) [49–52]. The anti-proliferative effects of BD and IC_50_ values on the aforementioned *in vitro* cancer cell lines and *in vivo* cancer models are summarized in Table 1 and Table 2, respectively. It has been noticed that the anti-proliferative effect of BD varied in a time-dependent and dose-dependent manner in all of the experiments. Importantly, no significant toxicity of BD was observed against normal control cell lines.

**Table 1.** Details of the anticancer efficacy of BD in several human malignancies using *in vitro* models

**Cancer type**	**Cell lines**	**Anti-cancer efficacy**	**Mode of action**	**Concentration (μM)**	**IC_50_ (anti-proliferation)**	**References**
NSCLC	A549 NCI-H292	Anti-proliferative	↑ROS, ↑pJNK, ↑Apoptosis; ↑LC3-II, ↑Autophagy	0–40	A549: 17.89 (48 h) NCI-H292: 14.42 (48 h)	[28]
	A549 H460 PC9 H1975	Anti-proliferative	A549, H460: ↑pJNK, ↓Bcl-2, ↑BAX, ↑caspase 3 and PARP, ↑Apoptosis	0–12.5	A549: 0.6 (48 h) H460: 0.5 (48 h) PC9: 1.0 (48 h) H1975: 2.7 (48 h)	[41]
Pancreatic adenocarcinoma	PANC1 SW1990 CAPAN-1	Anti-proliferative	PANC1: ↑p38-MAPK, ↓Bcl-2, ↑BAX, ↑caspase 3 and 8	< 0.1–> 30	PANC1: 2.53 (72 h) SW1990: 5.21 (72 h) CAPAN-1: 1.35 (72 h)	[29, 37]
Chronic Myeloid Leukemia	K562	Anti-proliferative	↓pAKT and pERK; ↓Ψm, ↑caspase 3 and 9, ↑PARP	0–12	6.37 ± 0.39 (72 h)	[44]
TNBC	MDA-MB-231	Anti-proliferative Anti-invasive Anti-migration	↓PI3K, ↓pAKT, ↑E-cadherins, ↓vimentin and β-catenin, partial EMT reversal	0–100	5.84 (48 h); 2.364 (72 h)	[30]
Osteosarcoma	MNNG/HOS U-2OS MG-63 Saos-2	Anti-proliferative Anti-invasive Anti-migration Anti-CLC	↓pSTAT3, ↓Cyclin D1, CDK4, CDK2, ↑Apoptosis; ↓pSTAT3, ↓CD133, SOX2, Oct-4, Nanog	0–20	MNNG/HOS: 0.9 (48 h) U-2OS: 0.05 (48 h) MG-63: 0.65 (48 h) Saos-2: 0.51 (48 h)	[47]
Hepatocellular carcinoma	Huh7 Hep3B	Anti-proliferative	↑Proteasome, ↓Total β-catenin, ↓Active β-catenin, ↓JAG1, ↓NICD, ↑Apoptosis	0–20	Approx. 2.5 (48 h)	[45]
	Bel7404 HepG2 Hep3B Huh7 PLC	Anti-proliferative	↓miR-95, ↑CUGBP2, ↑Apoptosis	0.25–1.5	Bal7407: ~1.0 (72 h) HepG2: ~0.8 (72 h) Hep3B: ~0.75 (72 h) Huh7: ~0.6 (72 h) PLC: ~0.8 (72 h)	[46]

Ψm: mitochondrial membrane potential; CD133: prominin-1; CDK2: cyclin dependent kinase 2; CUGBP2: Elav-like family member 2; JAG1: Jagged1; LC3-II: autophagy marker; Nanog: homeobox protein NANOG; NICD: cleaved intracellular domain of Notch receptor; Oct-4: octamer-binding transcription factor 4 (also known as POU5F1); PARP: poly (ADP-ribose) polymerase; p38-MAPK: p38 mitogen-activated protein kinases; pAKT: phosphorylated protein kinase B; pERK: phosphorylated extracellular signal regulated kinase; pJNK: phosphorylated c-Jun N-terminal kinase; pSTAT3: phosphorylated signal transducer and activator of transcription 3; SOX2: SRY (sex determining region Y)-box 2; CDK4: cyclin-dependent kinase 4; EMT: epithelial-mesenchymal transition; miR-95: microRNA-95

**Table 2.** Details of the anticancer efficacy of BD in several human malignancies using *in vivo* models

**Cancer type**	**Model used**	**Dose**	**Duration**	**Measurement frequency**	**Route of administration**	**Observed effects**	**Mode of action**	**References**
NSCLC	A549 cells in BALB/c-nu mice	40 mg/kg/day	15 days	Every 2 days	Intraperitoneal injection	↓Tumour growth	↑pJNK, ↑caspase 9 ↑Apoptosis; ↑LC3-II, ↑Autophagy	[28]
Osteosarcoma	MNNG/HOS cells in BALC/c-nu mice	2.5 mg/kg/2 days; 5.0 mg/kg/2 days	12 days	Every 2 days	Intraperitoneal injection	↓Tumour size ↓Tumour weight	↓pSTAT3, ↓MMP2 and MMP9, ↓Ki67	[47]
Hepatocellular carcinoma	Huh7 cells in BALC/c-nu mice	0.75 mg/kg/day; 1.5 mg/kg/day	10 days	Daily	Intravenous tail vein injection	↓Tumour growth	↑Proteasome, ↓Total β-catenin, ↓Active β-catenin, ↓JAG1, ↓NICD, ↑Apoptosis	[45]

MMP2: matrix metalloproteinase-2 (gelatinase A); MMP9: matrix metallopeptidase 9 (gelatinase B)

### Effect of BD against triple-negative breast cancer (TNBC)

TNBC is characterized by the absence or down-regulation of estrogen receptors, progesterone receptors, and human epidermal growth factor 2 receptors [53–55]. Due to its aggressiveness, malignancy, and distant recurrence, TNBC is usually accompanied by a poor clinical prognosis and short life expectancy [55, 56]. To date, the best therapeutic treatment for TNBC is restricted only to chemotherapy, surgical tumor removal, and limited clinical drugs which are still being evaluated in clinical trials [55, 57–59]. It is therefore imperative to uncover new sources of adjuvant or stand-alone treatment for TNBC, be it to combat TNBC or to alleviate discomfort from standard chemotherapy and surgical procedures. Luo et al. [30], have investigated the inhibitory effects of BD on MDA-MB-231 (TNBC) cells. They investigated the role of PI3K/AKT signaling in tumor metastasis with low doses of BD that does not affect cell viability as it was previously reported that the PI3K/AKT signaling pathway was implicated, albeit in colorectal cancer cells [60]. It was found that BD significantly decreased PI3K expression and AKT phosphorylation in a dose-dependent manner while total AKT levels remained constant, leading them to conclude that the PI3K/AKT signaling pathway was implicated in tumor metastasis of TNBC. While the role of PI3K/AKT signaling in cell proliferation was not clearly established in the study, it has long known that the PI3K/AKT signaling pathway regulates cellular proliferation, cell cycle, and apoptosis. Its dysregulation is also implicated in various *in vitro* cancer models, where PI3K and it’s downstream molecular targets are constitutively active, leading to uninhibited cell proliferation and cell growth [61]. Further, it was also found that the PI3K/AKT/mTOR pathway is frequently activated in TNBC [62]. With knowledge from existing literature, there is a high possibility that anti-proliferative and pro-apoptotic effects of the BD on TNBC could be mediated through the PI3K/AKT pathway. However, we can only hypothesize at best since it was never explicitly investigated in the context of proliferation and apoptosis.

### Effect of BD against pancreatic adenocarcinoma

Pancreatic adenocarcinoma comprises 95% of the diagnosed pancreatic cancer cases, with other cases being rarer forms of neuroendocrine cancers [63, 64]. Patients with pancreatic cancer have worse prognosis due to its aggressive, metastatic, and drug-resistance nature [65, 66]. Chemotherapeutic agents such as gemcitabine, 5-fluorouracil, and capecitabine are commonly administered for patients with pancreatic adenocarcinoma. To date, gemcitabine remains the chemotherapeutic agent of choice [64, 67], but acquired-gemcitabine resistance of pancreatic adenocarcinoma cells is the unsolved mystery [68–70]. Natural bioactive compounds like camptothecin (DNA topoisomerase I poison) could also be used as a chemotherapeutic agent but it showed only low therapeutic efficacy with severe toxicity [71]. It has been previously demonstrated that *Brucea javanica* fruit extract was able to exert cytotoxic and pro-apoptotic effects against pancreatic adenocarcinoma cell lines PANC1, SW1990, and CAPAN-1 [37, 72]. In a follow-up study in 2009 by the same team, BD was used directly instead of *Brucea javanica* fruit extract. It was shown that there was a BD dose-dependent apoptogenic effect on PANC-1 cells following Annexin V-PI double staining. Western blot analysis was also congruent with the results of the Annexin V-PI assay, whereby induction of caspase 3 and 8, enhancement of pro-apoptotic protein Bak, and decrease in anti-apoptotic protein Bcl-2 were seen. Further investigation elucidated that BD-induced cellular apoptosis was mediated through the necessary activation of the p38-MAPK signaling pathway as prior incubation with p38-MAPK inhibitor SB203580 attenuated caspase activation and DNA fragmentation.

MAPK participates in signaling pathways that are crucial for the maintenance of normal cellular processes like cell-cycle progression, cell proliferation, and apoptosis [73–77]. p38-MAPK constitutes one of the four subgroups within the MAPK family and respond to a wide range of extracellular stimuli such as stress or growth factor stimulation [78]. It is known that p38-MAPK activation could induce apoptosis and cell-cycle arrest, leading to tumor suppression. Conversely, inactivation, or down-regulation of p38-MAPK increases the tumorigenic potential of cells as DNA damage accumulates with each successive cell cycle [79]. Downstream molecular targets of p38-MAPK include c-Jun, signal transducers and activator of transcription 1 (STAT1), and p53 proteins amongst an abundance of other proteins, all of which are involved in regulating the balance between cellular proliferation and apoptosis [79–84].

It was proposed in the study that BD could have participated in both extrinsic and intrinsic apoptotic pathways. In the intrinsic pathway, BD would have acted as an extracellular stress signal which activates the p38-MAPK pathway. In the extrinsic pathway, BD would have acted as a ligand to membrane death receptors. No discrimination was made between those two apoptotic pathways when it came to examining the expression of caspase 3, caspase 8, and caspase 9 is known to be implicated in both mitochondrial-mediated apoptosis and receptor-mediated apoptosis [85].

### Effect of BD against chronic myeloid leukemia

Chronic myeloid leukemia is a myeloproliferative malignancy that is usually caused by a chromosomal rearrangement event between chromosome 22 and chromosome 9 to form the Philadelphia chromosome [86, 87]. It is characterized by over-expression of a fusion oncoprotein BCR-ABL1 that acts as a constitutively active defective tyrosine kinase, implicating downstream signaling pathways such as PI3K/AKT/mTOR, Janus kinase (JAK)/STAT and Ras protein family (Ras)/mitogen-activated protein kinase kinase (MEK), all of which are crucial in maintaining normal cellular proliferation and apoptosis [86, 88]. Conventional therapeutic agents like imatinib, nilotinib, dasatinib, ponatinib, and bosutinib, therefore, act as tyrosine kinase inhibitors to prevent constitutive activation of the receptor tyrosine kinases in CML [86, 89–91].

BD was found to induce cellular apoptosis via the intrinsic mitochondrial-apoptotic pathway to K562 cells in a time-dependent manner [44, 80]. The intrinsic apoptosis pathway is characterized by a loss of membrane potential leading to the release of cytochrome c (cyt c) to the cytosol. Cyt c then binds to apoptotic peptidase activating factor 1 and caspase-9 to form an apoptosome, which cleaves caspase 3 to trigger apoptosis [44, 92]. It was postulated that the PI3K/AKT and Ras/Raf/extracellular signal-regulated kinase (ERK) pathways were upstream targets of BD in the study. Indeed, phosphorylated AKT and phosphorylated ERK levels were attenuated in cells treated with BD. However, since ERK activation need not always lead to pro-survival signals as ERK activation was reported to exhibit pro-apoptotic functions [93, 94], ERK activity must be interpreted in context. It remains unclear whether BD acted as a receptor tyrosine kinase inhibitor prior to PI3K and ERK activation or as an activator of phosphatases to PI3K and ERK.

### Effect of BD against NSCLC

NSCLC constitute the majority of lung cancer cases in the world [95–100]. The effects of BD on the NSCLC cell lines A549, NCI-H292, and H460 has been investigated by different groups and reported that BD-induced intrinsic cellular apoptosis was mediated through the MAPK/JNK pathway [28, 42, 43]. Similar to p38-MAPKs, JNKs belong to the MAPK superfamily and regulate important biological processes like cellular proliferation and apoptosis. Phosphorylation of JNK will translocate it to the nucleus, where it phosphorylates c-Jun and forms activator protein 1, a transcription factor that is involved in the expression of pro-apoptotic proteins in both extrinsic and intrinsic apoptosis pathways [101]. In addition to apoptosis, BD was able to enhance autophagic flux in cell lines A549 and NCI-H292, another cellular process that can inhibit tumorigenesis and cancerous cell proliferation [28, 43].

Furthermore, it was highlighted that JNK activation and subsequent cellular apoptosis and autophagy was mediated mostly through BD-induced reactive oxygen species (ROS) production. Pre-treatment of cells with N-acetylcysteine, an antioxidant, before BD administration abolished apoptosis and autophagy almost entirely [28]. ROS are constantly being produced by cellular metabolic processes, and they are removed by antioxidant proteins [102]. Failure to maintain the dynamic equilibrium between ROS production and ROS elimination would result in oxidative stress to the cell [102, 103]. To this effect, ROS can activate different MAPK pathways to elicit different cellular responses. Herein, it is widely recognized that ROS oxidizes Trx (an antioxidant protein) to dissociate from apoptosis signal-regulating kinase 1 (ASK-1) (MAP3K5, another member of the MAPK family). The active ASK-1 would then activate downstream JNK pathways [102, 103]. BD also demonstrated the same effects *in vivo*, at least for A549 and NCI-H292 cell lines [28, 43].

### Effect of BD against osteosarcoma

Osteosarcoma is a relatively rare type of primary bone malignancy that occurs mostly during adolescence and young adulthood [104]. While tumors can be treated efficiently with traditional chemotherapy, increasingly resistant cancer stem cells (CSCs) pose a significant threat to recurrent cancer progression [104]. This highlights the need to develop agents that could target CSCs specifically in addition to proliferating tumors [105–108]. In this light, the effect of BD against osteosarcoma cell lines MNNG/HOS and U-2OS was explored by Wang et al. [47]. It was demonstrated that BD-induced inhibition of cellular proliferation was through modulating key proteins involved in cell cycle progression and promoting apoptosis. There was significant downregulation of Cyclin D1, CDK4, and CDK2 expression. Cyclin D1 is vital in regulating the transition from G1 to S phase, and over-expression of cyclin D1 has been associated with tumorigenesis [47]. Increased proapoptotic protein expression was also detected for cells treated with BD. The repression of the JAK2/STAT3 signaling pathway was seen to be implicated for cells with BD treatment, with particular attention being paid to the upregulation of phosphatase Tyrosine-protein phosphatase non-receptor type 6 (SHP1) that negatively regulates STAT3 [47].

**Figure 2.**
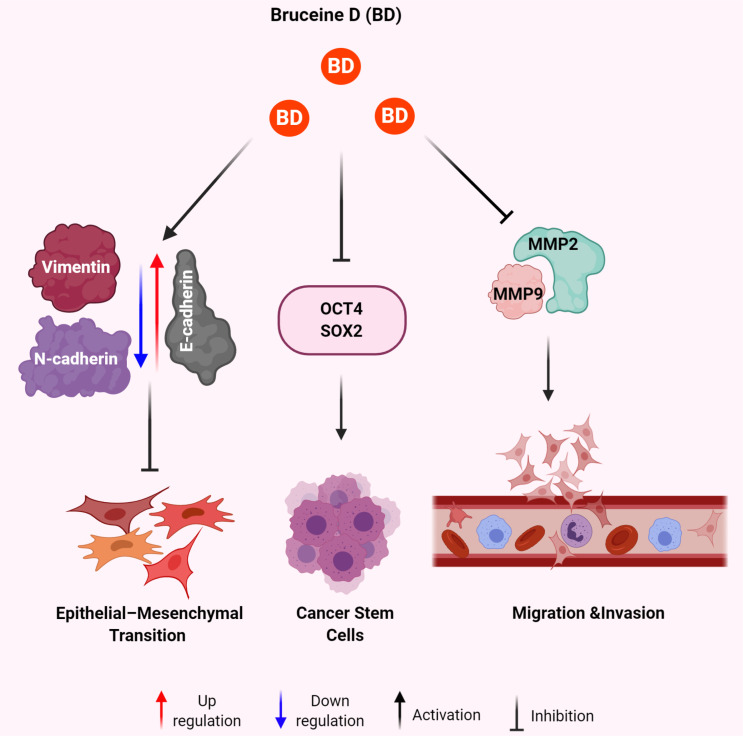
Mechanisms of BD for anti-metastatic effect in human malignancies. BD results in increased expression of E-cadherin and decreased expression of vimentin and N-cadherin leading to decreased EMT. BD treatment leads to decreased expression of OCT4 and SOX2 to eradicate the CSCs. BD exposure attenuates the expression of MMP2 and MMP9 to inhibit migration and invasion of cancer cells

Most notably however was that BD treatment was able to decrease the proportion of stem-like osteosarcoma cells and impaired the self-renewal ability of osteosarcoma stem cells [47]. This was quantified through the downregulation of biomarkers via flow cytometry and Western blot analysis of multiple CSC markers like CD133 and stem cell markers like SOX2, Oct-4, and Nanog (Figure 2) [47].

### Effect of BD against hepatocellular carcinoma (HCC)

HCC is one of the most common malignancies in the world [109–111]. To date, only a few targeted therapies have found to be effective against HCC and natural products may also exhibit their diverse anti-cancer actions against this malignancy [112–114]. Hence, there is a need for the development of cheaper and more encompassing therapeutic options. Two modes of action of BD-induced inhibition cellular proliferation were uncovered. In one study, BD suppresses proliferation via β-catenin/JAG1 pathways [45]. BD was demonstrated, both *in vivo* and *in vitro*, to induce proteasomes that degraded β-catenin and active β-catenin and pro-survival proteins from the transcriptional activities of active β-catenin from the canonical Wnt/β-catenin signaling pathway [45], which includes the JAG1 protein [115]. JAG1 in turn acts as a ligand to the canonical Notch signaling pathway, in which over-expression of JAG1 and/or aberrant activation of the Notch pathway is associated with cancer [116].

In a separate study using Hep3B, PLC, HepG2, Huh7, and Bel7407, BD suppressed cellular proliferation via miR-95 expression and induction of pro-apoptotic protein CUGBP2 [46]. There is growing evidence that miRNAs are capable of effecting oncogenic or tumor-suppressive functions, dysregulation of which have significant influence over tumorigenesis and its underlying cellular processes [117]. The oncogenic function of miR-95 is implicated in a wide range of cancers like osteosarcoma, recurrent NSCLC, and HCC, where down-regulation of miR-95 has been found to suppress tumor growth [38, 118]. BD was suggested as a negative regulator of the promoter of miR-95, whereby BD treatment decreased miR-95 expression. Using bioinformatics analysis via TargetScan and PicTar, it was further identified that the 3’ untranslated region of CUGBP2 mRNA was a direct downstream target for miR-95, and miR-95 binding interfered with CUGBP2 translation [119]. While CUGBP2 was previously shown to induce apoptosis in colon cancer cells via active stabilization and translation inhibition of pro-survival protein Mcl-1 [120], the mechanism of CUGBP2 within HCC has not been elucidated.

## Anti-metastatic effects of BD

Metastasis can be defined as the spread of neoplastic tissue to organs and systems beyond the origin of a benign tumor, usually resulting in the formation of new tumors [121–123]. It is an extremely complex process that involves a sequential series of steps known as the invasion-metastasis [124–129]. In brief, metastasis begins with the invasion of cancer cells into the local extracellular matrix, followed by penetration of endothelium basal membrane and entry into blood vessels and/or lymph nodes, usually ending with extravasation into the surrounding tissue of a distant organ [128–133]. In particular, it was proposed that EMT is paramount to the start of the cascade. It is worth noting that EMT is not exclusive only to tumorigenesis as it also participates in many other normal cellular processes like embryonic development, wound healing, tissue regeneration, and fibrosis [127, 134–136]. However, when EMT is reactivated (partially or fully) in cancer cells indiscriminately, these cells acquire mobility, a mesenchymal feature that augments invasiveness and metastasis of cancer cells [128, 136, 137]. Through extensive research of *in vitro* cancer cell-based models, it has been found that the hallmarks of EMT are overexpression of EMT regulatory transcriptional factors such as the family of zinc-finger proteins (SNAIL)1/2 and Twist-related proteins (TWIST)1/2 as well as dysregulation of key proteins like E-cadherin, N-cadherin, vimentin, and β-catenin (Figure 2) [128, 136, 138, 139]. The anti-metastatic effects of BD have only been studied in human TNBC and osteosarcoma. In a study by Luo et al. [30], have used low concentrations of BD (1 μM to 4 μM) to determine its effect on the migration and invasion of breast cancer cells. This study displayed that MDA-MB-231 cells treated with BD had lowered migratory and invasive capabilities which varied in a dose-dependent manner [30]. It was also observed that loss of E-cadherin and overexpression of vimentin and β-catenin was abrogated in cells treated with BD varying in a dose-dependent manner, suggesting that the EMT program was successfully reversed, if not partially, in MDA-MB-231 cells (Figure 2). In another study, it was reported that BD-treated osteosarcoma cell lines MNNG/HOS and U2-OS displayed reduced expression of N-cadherin, MMP-2, and MMP-9, all of which are EMT biomarkers and play important roles in tumorigenesis [47]. Wang et al. [47], took it one step further and exemplified the partial involvement of the JAK2/STAT3 signaling pathway in BD-induced inhibition of osteosarcoma growth and migration. Constitutively active STAT3 has been shown to induce tumor formation in osteosarcoma. In both cell lines, phosphorylated JAK2 and STAT3 was decreased while phosphatase SHP1, a negative regulator of STAT3, was upregulated, indicating that BD reversed the constitutive activation of the JAK2/STAT3 signaling pathway in the osteosarcoma cell lines [47].

Although the anti-metastatic effects of BD seem promising, both studies only demonstrated the effect of BD against their respective cancer cell lines *in vitro*. *In vitro*, modeling can model certain aspects of tumorigenesis such as cellular migration and invasion, but it suffers from certain inherent limitations as it lacks the complete physiological interactions *in vivo*. In order to fully assess the anti-metastatic effects of BD, *in vivo* animal models should be carefully examined before concluding.

## Potential anti-inflammatory effects of BD

Chronic inflammation has been hailed as one of the significant hallmarks of cancer progression and tumorigenesis. Through chronic inflammation, a conducive tumor microenvironment is formed from the plethora of inflammatory cells and cytokines available in the vicinity, promoting tumor survival and proliferation[140–142]. One of the prominent players involved in the inflammation-tumorigenesis cross-talk is nuclear factor-κB (NF-κB). NF-κB is a nuclear transcription factor that regulates genes responsible for the body’s immune responses as well as other essential physiological responses like inflammation, cell proliferation, and apoptosis [143, 144]. BD has been observed to be the second most potent inhibitor of inflammation in rodents, with the first being brusatol, another quassinoid extracted from *Brucea javanica* as well [145]. Recently, the anti-inflammatory properties of *Brucea javanica* oil emulsion (BJOE) in dextram sulfate sodium-induced ulcerative colitis has been investigated [146]. Oleic acid and linoleic acid have been found to have major components of BJOE, and that both oleic acid and linoleic acid have been shown to display anti-inflammatory properties. Despite BD not being a component of BJOE, it was found that the NF-κB pathway was significantly attenuated via inhibition of NF-κB and nuclear factor of kappa light polypeptide gene enhancer in B-cells inhibitor, alpha (IκBα). Another study on ulcerative colitis directly used BD in 0.5% sodium carboxymethyl cellulose solution and BD delivered using a self-nanoemulsifying drug delivery system (BD-SNEDDS) [146, 147]. It was reported that in addition to attenuating the NF-κB pathway, BD was also capable of suppressing cyclooxygenase-2, an enzyme implicated in inflammation [18]. In a separate study on Parkinson’s disease, BD was also found to inhibit inflammation as well albeit via the nuclear factor erythroid 2-related factor 2 (Nrf2) signaling pathway.

Although there is currently no evidence of BD’s anti-inflammatory properties in cancer models, given the highly intertwined nature of NF-κB in the cross-talk between inflammation and cancer, it would therefore not be surprising if BD exhibited anti-inflammatory properties in inhibiting tumorigenesis and cancer development.

## Pharmacokinetics, toxicity and metabolism

As mentioned previously, BD was explored as an anti-inflammatory agent to ulcerative colitis. The pharmacokinetics of BD-suspension in 0.5% sodium carboxymethyl cellulose solution was only established in the same ulcerative colitis murine model study relative to BD-SNEDDS. The concentration used for both BD and BD-SNEDDS was 3.0 mg/kg^–1^.

As established by the aforementioned BD research, all of them noted non-significant toxicity against normal cell lines *in vitro* (cell viability maintained) and *in vivo* (maintenance of growth parameters like weight). There is currently no study indicating any other toxicity, allergies, or contraindications when using BD. Drug metabolism of BD *in vitro* and *in vivo* and whether any adverse metabolites are produced remain unknown.

## Clinical application of BD

Currently, BD is commonly used with other quassinoids and bioactive compounds within BJOE as adjuvant therapy to chemotherapy and radiotherapy of various malignancies in China, some of which include brain cancers, gastrointestinal cancers, and urological malignancies. BJOE is administered for its anticancer effects and/or attenuation of side effects from conventional cancer treatment [26]. Clinical trials involving BD as a stand-alone treatment option or adjuvant therapy had yet to be conducted. Further research on its clinical use should, therefore, be conducted to fully ascertain its inhibitory effects on tumorigenesis in human patients.

## Conclusion and future directions

This review highlights the antineoplastic effects of BD in various cancer models. The BD has been demonstrated to exhibit anti-proliferative and pro-apoptotic effects against various cancer cell lines via inhibition of the key regulatory signaling pathways like PI3K/AKT/mTOR, JAK/STAT, JAG1/Notch. Also, there were promising metastatic regulatory effects on breast cancer and osteosarcoma cell lines and potential anti-inflammatory effects. However, there is still much to be learned about the exact molecular targets of BD as many of the studies were mainly concerned with finding its antineoplastic effects and not its direct molecular target *per se*. Given the mounting evidence of BD efficacy against various cancer cell lines *in vitro* and *in vivo*, BD could potentially be considered as yet another novel anticancer drug and a future candidate for clinical trials and development. In the future, the combination of BD with standard chemotherapeutic drugs may be explored for developing better treatment options with the aim of long-term disease-free survival.

## Abbreviations

AKT: protein kinase B
ASK-1: apoptosis signal-regulating kinase 1
BAX: Bcl-2 associated protein
Bcl-2: B-cell lymphoma 2
BD: bruceine D
BD-SNEDDS: BD-loaded self-nanoemulsifying drug delivery system
BD-suspension: BD suspended in 0.5% sodium carboxymethyl cellulose solution as control
BJOE: *Brucea javanica* oil emulsion
CD133: prominin-1
CDK2: cyclin-dependent kinase 2
CDK4: cyclin-dependent kinase 4
CSCs: cancer stem cells
CUGBP2: Elav-like family member 2
cyt c: cytochrome c
EMT: epithelial-mesenchymal transition
ERK: extracellular signal-regulated kinase
HCC: hepatocellular carcinoma
JAG1: Jagged1
JAK: Janus kinase
JNK: c-Jun N-terminal kinase
LC3-II: autophagy marker
MAPK: mitogen-activated protein kinases
Mcl-1: myeloid cell leukemia 1
MEK: mitogen-activated protein kinase kinase
miR-95: microRNA-95
MMP2: matrix metalloproteinase-2 (gelatinase A)
MMP9: matrix metallopeptidase 9 (gelatinase B)
mTOR: mammalian target of Rapamycin
Nanog: Homeobox protein NANOG
NF-κB: Nuclear factor-κB
NICD: the cleaved intracellular domain of Notch receptor
NSCLC: non-small cell lung cancer
Oct-4: octamer-binding transcription factor 4
p38-MAPK: p38 mitogen-activated protein kinases
pAKT: phosphorylated protein kinase B
PARP: poly (ADP-ribose) polymerase
pERK: phosphorylated extracellular signal-regulated kinase
PI3K: phosphatidylinositol 3-kinase
pJNK: phosphorylated c-Jun N-terminal kinase
pSTAT3: phosphorylated signal transducer and activator of transcription 3
Ras: Ras protein family
ROS: reactive oxygen species
SHP1: Tyrosine-protein phosphatase non-receptor type 6
SOX2: SRY (sex-determining region Y)-box 2
STAT: signal transducers and activator of transcription
TNBC: triple-negative breast cancer
Ψm: mitochondrial membrane potential

## Declarations

### Copyright

© The Author(s) 2020.
